# *PLOS Medicine* 2014 Reviewer Thank You

**DOI:** 10.1371/journal.pmed.1001806

**Published:** 2015-02-27

The Public Library of Science (PLOS) and the *PLOS Medicine* editorial team would like to warmly thank all those individuals who participated in the peer review process this past year at *PLOS Medicine*. During 2014 over 900 reviewers from around the world helped evaluate the 194 articles we published, and offered valuable input and advice for those authors whose papers we did not publish.

The names of our 2014 reviewers are listed in S1 Reviewer List. We are grateful to all our reviewers for sharing their expertise with *PLOS Medicine* authors, and for helping us to publish a highly read and highly regarded open access medical journal.

## Supporting Information

S1 Reviewer List Alphabetical list of 2014 *PLOS Medicine* reviewers. (PDF)
