# Supplementary material for: PLOS Medicine 2014 Reviewer Thank You
Source: PLoS Med. 2015 Feb 27;12(2):e1001806. doi: 10.1371/journal.pmed.1001806 (PMC4344346; doi:10.1371/journal.pmed.1001806)
Supplement: S1 Reviewer List — (PDF) [file pmed.1001806.s001.pdf]

PLOS | MEDICINE

PLOS Medicine would like to thank all those who reviewed on behalf of the journal in 2014:

Rasmus á Rogvi
Ain Aaviksoo
Gilbert Abiiro
Seye Abimbola
Laith Abu-Raddad
Gareth Ackland
John Adams
Adedeji Adebayo
Ifedayo Adetifa
Rafi Ahmed
Ali Ahmed
Arihiro Aihara
G. Caleb Alexander
Ryan Allen
Stefan Ambs
Alice Ammerman
Fiifi Amoako Johnson
Sonia Anand
Jason Andrews
Maria Aranda
Elise Arrivé
Anthony Avery
Helen Ayles
Soraya Azari
Till Bärnighausen
Paluku Bahwere
Frank Baiden
John Balmes
Jay Baraban
Chris Barker
Ronald Barr
Aluisio Barros
Stefania Basili
Ingrid Bassett
Andrew Bastawrous
Hilda Bastian
Brian Bateman
Carine Baxerres
Andrew Beck
James Beeson
Chaim Bell
Derek Bell
Eran Bendavid
Valerie Beral
Jonas Bergh
Olivier Berton
Lars Bertram
Philippe Beutels
Chris Beyrer
Kavi Bhalla
Sonia Bhalotra
Prashant Bharadwaj
Sohinee Bhattacharya
Achuyt Bhattarai
Deborah Billings
Oleg Bilukha
Christine Biron
Paul Birrell
Zeno Bisoffi
Michael Blaha
Annalisa Blasetti
Catherine Blish
Delia Boccia
Michael Boele van Hensbroek
Christophe Boete
Knut Borch-Johnsen
Michiel Bos
Jizzo Bosdriesz
Isabelle Boutron
Jack Bowden
Anna Bowen
Elizabeth Bradley
Paul Brand
John Britton
Clarissa Brocklehurst
Jürgen Brockmöller
Kristina Broliden
Göran Broström
Jennifer Bryce
John Buchan
Jesse Bump
Roberto Burioni
Frederick Burkle, Jr
Paulo Buss
Peter Byass
Helen Bygrave
Joanne Cacciatore
Sandy Cairncross
Suzanne Cannegieter
Simon Capewell
Waldemar Carlo
David Carpenter
Juan Carrero
Richard Chaisson
William Checkley
Bernard Cheung
Gerardo Chowell
Kathryn Chu
Brian Claggett
Thomas F. Clasen
Archie Clements
Frank Cobelens
Jeff Collin
Jeannie Collins

Margaret Conner
Elizabeth Corbett
Marilys Corbex
Rejane Corrêa Marques
Michel Cot
Benjamin Cowling
Peter Croft
Russ Cucina
Luis Cuevas
Oliver Cumming
Steve Cunningham
Kathleen Curtis
Francois Dabis
Philipp Dahm
Benjamin Dalziel
Sharon Daniel
Derek Daniels
Karen Daniels
Mary-Ann Davies
J. Lucian Davis
Natalie Dayan
Richard Daynard
Manuela De Allegri
Paul de Bakker
Nihal de Lanerolle
Tulio de Oliveira
Martin de Smet
Anneke de Vos
Louisa Degenhardt
Nikos Demiris
Antoine Depaulis
Don Des Jarlais
Puneet Dewan
Michael Dewey
Mehul Dhorda
Maximilian Diehn
D.B. Diercks
Wolfram Doehner
Colin Dollery
Arjen Dondorp
Christl Donnelly
Danny Dorling
Grant Dorsey
Delanyo Dovlo
Cinthia Drachenberg
Alison Drake
Peter Drobac
Chris Dulla
Mignon Duplessis
Jill Durocher
Jeff Eaton
Matthias Egger
Julian Elliott
David Ellwood
Jonathan Emberson
Claudia Emerson
Mike English
Cyril Engmann
David Epstein
Aneez Esmail
Mark Espeland
Nir Eyal
Kristine Færch
Christopher Fairley
Jordan Feld
Ricardo Fernandes
Rashida Ferrand
David Fidler
David Fidock
Katherine Fielding
Eric Finkelstein
Thomas Finucane
James Fleet
Nancy Fleischer
Vicki Flenady
Peter Flom
Bruce Foggo
Simon Foote
Nathan Ford
Edward Fottrell
Silvia Franceschi
Rachel Freathy
Matthew Freeman
Atle Fretheim
Thomas Friedrich
Nial Friel
Adriane Fugh-Berman
Barbara Fuhrman
Jennifer Furin
Marc-Andre Gagnon
Emmanuela Gakidou
Omar Galárraga
Patricia Galipeau
Giovanni Gambassi
Rajiv Gandhi
Sam Gandy
Wei Gao
Jason Gardosi
Richard Garfield
Paul Garner
Coral Gartner
Piers Gatenby
Bradley Gaynes
Stacie Geller
Holger Gerhardt
Peter Gething
Dirk Gevers
Cyrus Ghajar

Davina Ghersi
John Gimnig
Stanton Glantz
Richard Glassock
Paul Glasziou
Paul Goepfert
Ameena Goga
Nick Golding
Beatrice Golomb
Jonathan Golub
Raquel Gonzalez
Steve Goodacre
Myriam Gorospe
Amanda Graf
Barney Graham
Rebecca Grais
Philippe Grandjean
Nicholas Grassly
Andrew Gray
Nicola Greenlaw
Gregg Greenough
Brian Greenwood
Kimberly Gregory
David Grelotti
Jamie Griffin
Paul Gringras
Alden Gross
Ron Grunstein
Michel Guillot
Roman Gulati
Yuming Guo
Rajesh Gupta
David Gwatkin
Holly Hagan
Gareth Hagger-Johnson
Simon Hales
Robert Hall
Wayne Hall
Michael Hallek
Timothy Hallett
Tomas Hanke
Catherine Hankins
Ashraful Haque
Stephan Harbarth
Sam Harper
Steve Harris
Omar Hasan
Karin Hatzold
Derek Hausenloy
David Hayes
Iona Heath
Jennifer Hellier
Sean Hennessy
Jay Herman
Miguel Hernan
Adrian Hernandez
James Herrington
Adam Hersh
Manuel Hetzel
Matthew Hickman
Lauri Hicks
Lanis Hicks
Madelyn Hicks
Suzanne Hill
Philip Hill
Andrew Hill
Hans Hirsch
Marcia Hobbs
David Hockenbery
T. Deirdre Hollingsworth
Lars Holmberg
Jaco Homsy
Yuling Hong
Jan Hontelez
Thomas Hope
Guy Howard
Alexandra Howell
Kelly Hsieh
Chung-Cheng Hsieh
Kam Hui
Wen Hung Chung
Myriam M.G. Hunink
Paul Hunter
Lisa Hurt
Barry Hurwitz
W. Huskins
Jennifer Hutcheon
Benedikt Huttner
Andrew Hyland
Fumiaki Imamura
Miren Iturriza-Gómara
Rebecca Ivers
Debra Jackson
Christopher Jackson
Steven Jacobson
Michael Jacobson
Shabbar Jaffar
W. Philip James
William Jeffcoate
Neil Jenkins
Rachel Jenkins
Michael Jerrett
Zhenghui Jiang
Eliana Jimenez-Soto
Steven Joffe
Leigh Johnson
Allan Johnson
Grace John-Stewart

Rohina Joshi
Kaumudi Joshipura
Yuh-Shan Jou
Min Jun
Annemarie Jutel
Ephata Kaaya
Joseph Kagaayi
James Kahn
Malek Kamoun
Steve Kanters
David Kaplan
Nestor Kapusta
Stephanie Karst
Martijn Katan
Clare Kelleher
Scott Kellerman
James Kellner
Andre Kengne
Marko Kerac
Kate Kerber
Meeta Kerlin
Aaron Kesselheim
Ayesha Kharsany
Saye Khoo
Kamlesh Khunti
Peter Kilmarx
Marissa King
Michael King
Donald Klepser
Keith Klugman
Marian Knight
Juhani Knuuti
Isaac Kohane
Jillian Kohler
Kairi Kolves
James Koopman
Jeffrey Koplan
Roger Kouyos
Daniel Kramer
Katharina Kranzer
Tamara Kredo
Margaret Kruk
Ritushree Kukreti
Daniel Kuritzkes
Margot Kushel
Tezer Kutluk
Pawan Labhasetwar
Nicos Labropoulos
Carl Lachat
Oliver Laeyendecker
Louise Lafortune
Emmanuel Lagarde
Pagona Lagiou
Richard Laing
David Lalloo
Jon Laman
Claudia Langenberg
Bruce Lanphear
Samia Laokri
Elysia Larson
Mattias Larsson
Min-yu Lau
Michael Law
Michael Lebens
James LeCheminant
Dean Lee
Leonard Leibovici
Paul Leigh
Maria Lemos
Lindsey Lenters
Russell V. Lenth
Charles Leonard
Craig Leonardi
Justin Lessler
Joel Lexchin
Klaus Lieb
Jennifer Lin
Klaus Linde
Christopher Lippincott
Marc Lipsitch
Paul Little
Megan Littrell
Huw Llewelyn
Suzanne Lloyd
Barbara Lopes Cardozo
Michael T. Lotze
Nicola Low
David Lucas
Pisake Lumbiganon
Ronald Ma
David Mabey
Alan MacDonald
Sarah Macfarlane
Steven Mack
Nick Macklon
Malcolm Macleod
Freya MacMillan
Barbara Mahon
Jacqueline Major
Muhammad Mamdani
Melinda Manore
Ulrich Mansmann
Valeria Mas
Farrah Mateen
Colin Mathers
Diane Mathis
Fiona Matthews
Anne McCartt

James McCaw
Alex McConnachie
Ross McDevitt
Patricia McGettigan
Sean McGuigan
Martin McKee
Andrew McMichael
David McMurray
Gertjan Medema
Susan Meffert
Christoph Meier
Andreas Meisel
Yohannes Melaku
Erik Melen
Marc Mendelson
Martin Mengel
Bijoy Menon
Dick Menzies
William Miller
Grant Miller
Anthony Miller
Christopher Millett
Edward Mills
Philip Minor
Barbara Mintzes
Olivo Miotto
Sulma Mohammed
Ben Mol
Tracy Monk
Suerie Moon
Elinor Moore
Steven Moore
Ann Moormann
Ed Moran
Craig Morrell
Laust Mortensen
Joel Mossong
Peter Muennig
Robert S. Munford
Senthil Muthuswamy
John Myburgh
Landon Myer
Jean Nachega
Shigekazu Nagata
Nico Nagelkerke
Khurram Nasir
J. Craig Nelson
Eugene Nelson
Marion Nestle
Jennifer Neuman
Paul Newcombe
Marie-Louise Newell
Paul Newton
Ernest Ng
Mindie Nguyen
Mark Nicol
Markku Nieminen
Kosaku Nitta
Emmanuel Njeuhmeli
Abdisalan Noor
Jane Norman
Shane Norris
Kari North
Francois Nosten
Andrew Nunn
Ziad Obermeyer
Keelin O'Donoghue
John Oghalai
Fredros Okumu
Casey Olives
Wendy O'Meara
Gorik Ooms
Steven Opal
Kathleen O'Reilly
S. Owen
Kimberly Page
Franco Pagnoni
Madhukar Pai
Colin Palmer
Scott Palmer
Wen-Harn Pan
An Pan
William Pao
Shantini Paranjothy
Ira Pastan
Anushka Patel
Mical Paul
Melissa Pearson
Anne Peasey
Oluf Pedersen
Michael Peeters
Frederica Perera
Steffen Petersen
Ronald Petersen
Ruth Pfeiffer
Genevieve Pham-Kanter
Barbara Phillips
Elizabeth Phillips
Michael Pignone
Munir Pirmohamed
Taina Pitkäaho
Richard Pitman
Thomas Platts-Mills
Paul Poirier
Stephanie Polus
Roberto Pontremoli
Alan Poots
Esteban Porrini

Aidin E. Pour
William Powderly
John Powers
Andrew Prendergast
Ric Price
Holly Prigerson
Josef Priller
Martin Prince
Trevor Pugh
Rachel Pullan
Rajesh Puranik
Firdausi Qadri
Liqiang Qin
Alejandro Rabinstein
Michael Ramharter
Hilary Ranson
Sreenivasa Rao Kondapally Seshasai
Michael Rawlins
Joel Ray
Fahad Razak
Jennifer Read
Leanne Redman
Christiane Reitz
Jennifer Requejo
Frank Rhame
Maria Ribeiro
Peter Rice
Tom Richards
Pascal Richette
Joel Richter
Valéry Ridde
Steven Riley
Guus Rimmelzwaan
David Roberts
Mike Robson
William Rodriguez
Anne Rogers
Stephen Rogerson
Susannah Rose
Sydney Rosen
Nitzan Rosenfeld
Kenneth Rosenthal
Mary Jane Rotheram-Borus
Kenneth Rothman
Mark Rothstein
Sarah Rowland-Jones
Andrew Rule
Edward Ryan
Cecilia Söderberg-Naucler
Kalpana Sabapathy
Lora Sabin
Nandita Saikia
Ian Saldanha
Julia Samuelson
Peter Sandercock
Nick Sanders
Mary Sano
Naveed Sattar
Birgit Sawitzki
Saul Schaefer
Wolf-Peter Schmidt
Kathryn Schnippel
Matthias Schwab
Lisa Schwartz
Robert Schweitzer
Lesley Scott
Dinesh Selvarajah
Kwonjune Seung
Leigh Anne Shafer
Nathan Shaffer
Aziz Sheikh
Paul Shekelle
Bryan Shepherd
Sruti Shiva
David Sidransky
Frederick Sierles
Richard Simon
Cyrena Simons
Lone Simonsen
David Sinclair
Amit Singal
Mervyn Singer
Jerome Singh
Sodiomon Sirima
Jacek Skarbinski
Zili Sloboda
Peter Smith
Alexander Smith
Thomas Smith
Karen Smith-McCune
Josef Smolen
Harold Snieder
Heidi Soeters
Daniel Solomon
Harald Sontheimer
Aurélia Souares
João Paulo Souza
David Spiegelhalter
Glen Spielmans
R. Srinivasa Murthy
Cynthia Stanton
Pär Stattin
David Stephens
William Stones
Steffanie Strathdee
David Stuckler
David Studdert
Hugh Sturrock

Kanta Subbarao
Sandeep Subramanian
Jonathan Sugimoto
Amitabh Suthar
Maarten Taal
Shahrad Taheri
Bee-Choo Tai
Yemisi Takwoingi
Jeffrey Talbert
Kristina Talbert-Slagle
Teresa Tamayo
Joel Tarning
David Taylor-Robinson
Michael Templeton
Mihaela Teodorescu
Feiko ter Kuile
William Theodore
Grant Theron
Christoph Thiemermann
Paul Thomas
Brett Thombs
John Thompson
Mangesh Thorat
Nicholas Timpson
Martin Tobin
Mark Tomlinson
Toshifumi Tomoda
Stephen Tong
David Torgerson
Brian Trainor
Thach Tran
Caroline Trotter
Alexander Tsai
James Tumwine
Rebecca Turner
Kingsley Ukwaja
K.P. Unnikrishnan
Jennifer Utter
Matti Uusitupa
Catherine Valentine
Tom van der Poll
Jean-Pierre Van Geertruyden
Ingeborg van Geijlswijk
Thomas Vaughan
Effy Vayena
Ronald Veazey
Peter Ventevogel
Marcos Vera-Hernández
Sten Vermund
Cecile Viboud
Peter Vickerman
Andrew Vickers
Jean-Louis Vincent
Julian Vivian
Lorenz von Seidlein
Elizabeth Wager
Peter Waiswa
A. Sarah Walker
Jacco Wallinga
Xiao-Fan Wang
Christopher Ward
Nicholas Wareham
James Wason
Patrick Webb
Andrew Weeks
Daniel Weinberger
Bradley Weiner
Helen Weiss
H.G. Welch
Mary White
Neil White
Beate Wieseler
Joshua Willey
Hywel Williams
Rebecca Williams
Darrell Wilson
Adelaide Withers
Martin Wong
Robert Wood
Mark Woolhouse
Olivia Wu
Tangchun Wu
Maria Yazdanbakhsh
Weimin Ye
Kojo Yeboah-Antwi
John Yudkin
Basia Zaba
Anna Zajacova
